# The Efficacy and Effectiveness of Non-ablative Light-Based Devices in Hidradenitis Suppurativa: A Systematic Review and Meta-Analysis

**DOI:** 10.3389/fmed.2020.591580

**Published:** 2020-11-03

**Authors:** Abdulhadi Jfri, Anjali Saxena, Julie Rouette, Elena Netchiporouk, Augustin Barolet, Elizabeth O'Brien, Daniel Barolet, Ivan V. Litvinov

**Affiliations:** ^1^Division of Dermatology, McGill University Health Centre, Montreal, QC, Canada; ^2^Department of Epidemiology, Biostatistics, and Occupational Health, McGill University, Montreal, QC, Canada; ^3^Centre for Clinical Epidemiology, Lady Davis Institute, Jewish General Hospital, Montreal, QC, Canada

**Keywords:** hidradenitis suppurativa, lasers, hair removal, neodymium-doped yttrium aluminum garnet (Nd:YAG), alexandrite, intense pulse light (IPL), light-based devices

## Abstract

Hidradenitis suppurativa (HS) is a chronic inflammatory skin disorder that may be treated with non-ablative light-based devices; however, no systematic reviews on the topic exist to date. We conducted a systematic review and meta-analysis to determine efficacy of non-ablative light-based devices in treating HS. Specifically, a systematic review was conducted using MEDLINE, EMBASE, Web of Science and CINAHL. We analyzed the use of non-ablative light-based devices in the treatment of HS. At least two investigators performed title/abstract review and data extraction. Meta-analysis was conducted using comprehensive meta-analysis software. 5 RCTs and 11 case reports/series were included (*n* = 211 unique patients). No observational studies were found. For Nd:YAG laser, meta-analysis of 3 RCTs reported improvement in modified HS Lesion Area and Severity Index (HS-LASI) when compared to control subjects. In addition, three case reports/series reported HS-LASI, Physician Global Assessment (PGA) scores and number-of-lesion improvements in treated patients. For intense pulsed light (IPL), two RCTs reported HS-LASI and Dermatology Life Quality Index (DLQI) score improvements. For Alexandrite laser, one case report showed lesion improvement. In conclusion, meta-analysis of Nd:YAG laser in HS patients suggests significant improvement in HS-LASI scores. For IPL, evidence is limited, but suggests improvement in HS-LASI and DLQI scores. For Alexandrite laser, evidence precludes conclusions. Given small sample sizes and inconsistent reporting scales, larger RCTs are required to better determine the efficacy of these modalities in treating HS.

## Introduction

Hidradenitis suppurativa (HS) is a chronic inflammatory skin disease of intertriginous regions with a prevalence of 1 to 4% worldwide (1). It is thought to result from pilosebaceous unit occlusion and dilation, followed by follicular rupture, altered cytokine response, and abnormal microbiota in genetically predisposed individuals (2–4). Patients present with painful inflammatory papules and nodules that can progress to sinus tracts, hypertrophic, and keloid scars (5, 6). Lesions can be painful, disfiguring and malodorous leaving patients with depression and social isolation (7, 8). Smoking, obesity, and genetic factors are known risk factors for HS and likely play a role in its pathogenesis (2).

HS can be difficult to control depending on disease severity which is commonly classified by Hurley staging consisting of stages I (mild), II (moderate), and III (severe) (9). Mild disease is typically treated with topical and/or oral antibiotics (e.g., clindamycin). Moderate disease can be treated with intralesional corticosteroids, oral antibiotics (e.g., doxycycline, minocycline, rifamycin or clindamycin), retinoids (e.g., isotretinoin), hormonal medications (e.g., spironolactone) amongst other options. Advanced disease may require biologic therapies (e.g., high dose anti-TNF-alpha therapy), surgical deroofing or excision (10). The use of laser and other light-based devices in the treatment of HS has recently increased (11).

CO_2_ laser was the first to be studied in HS patients and was used as a surgical tool for deroofing and excision of HS sinus tracts (12, 13). Its cutting and vaporization ability has allowed for scar reconstruction with minimal bleeding (14). While fractionated CO2 lasers are used to surgically excise nodules and sinus tracts, non-ablative lasers and light therapies including neodymium-doped yttrium aluminum garnet (Nd:YAG) 1,064 nm (15), Alexandrite 755 nm (16, 17) and intense pulse light (IPL) (18) have shown benefits by targeting the hair follicle directly, destroying the pilosebaceous unit. This is intriguing given that the hair follicle element and the follicular inflammation are central to the pathogenesis of HS (4). The long-pulsed Nd:YAG and Alexandrite are non-ablative lasers that destroy the hair follicle by targeting melanin and water chromophores (15, 16).

Lasers emit light by amplifying photons optically based on electromagnetic radiation, and each photon is delivered at a precise vibrational state and power (17). In contrast, IPL emits broad wavelengths, using filters to narrow the spectrum. Lasers and IPL target (a) melanin (found abundantly in hair follicles leading to follicular necrosis) and (b) water molecules in the dermis, making both suitable treatment options for lighter-skin phototype HS patients (19), but despite their potential efficacy in treating HS, evidence of their actual effectiveness in case reports, case studies, and small randomized controlled trial (RCTs) (20) supporting their usage is limited.

Currently, only one systematic review exists providing a general overview on all lasers (ablative and non-ablative) in treating HS. None specifically evaluated the role of non-ablative light therapies and no meta-analysis has ever been conducted (21). We conducted the first systematic review and meta-analysis examining the evidence behind non-ablative light therapies (mostly light-based hair removal devices) in the treatment of HS. Given the significant costs of non-ablative light therapy, physicians recommending their use have an obligation to ensure that the theoretical potential of these treatments is supported by evidence. The results of this review suggest that with regards to therapeutic impact, ablative light hair removal tools are not only efficacious (have the potential to improve HS) but also effective (positive results demonstrated). What remains to be determined is whether this can be shown also for cost effectiveness.

## Materials and Methods

### Literature Search

This study was conducted in accordance with the Preferred Reporting Items for Systematic Reviews and Meta-Analyses (PRISMA) (22). MEDLINE, EMBASE, Web of Science and CINAHL were searched independently by two investigators (AJ, AS) from inception through April 2020. Search terms were “hidradenitis suppurativa,” “acne inversa,” “verneuil disease,” and “laser,” “intense pulse light,” “light.” No language restriction was applied.

### Eligibility Criteria

All study designs were eligible for inclusion (RCTs, observational studies, case series, and case reports). Review articles and articles discussing the use of conventional (normal mode) or fractional CO2 lasers for scars or surgery were excluded.

### Data Extraction

Data extraction was conducted by two independent reviewers (AJ, AS). Extracted data included: study design, number of patients, Fitzpatrick skin type, HS severity measured by Hurley staging, laser type [Nd:YAG 1,064 nm (15), Alexandrite 755 nm (16, 17), or IPL (18, 23)], laser characteristics (fluence (J/cm^2^), spot size (mm), pulse duration (ms).

### Quality Assessment

Risk-of-bias of included RCTs was assessed using the revised Cochrane risk-of-bias assessment version 2 (24), which is composed of five domains that assess risk of bias from initial randomization step through reporting step. Based on signaling questions, each domain was assigned an estimated risk-of-bias designated as “low,” “high,” or “some concerns.”

Case reports and case series were assessed using a published methodological tool for case reports and case series that provided scores for selection, ascertainment, causality, and reporting (25). Studies scoring 50% or more (4 or more “yes” answers) were considered valid.

### Outcomes Measures

The modified HS-LASI score (15) is composed of three physician-reported clinical components and four patient-reported symptoms. Clinical components were as follows: #1 lesion morphology: fistula 4 points, nodule 2 points, abscess and scar 1 point each; #2 distance between two lesions or size (if only one lesion): <5 cm, 2 points, 5–10 cm, 4 points, and >10 cm, 8 points; #3 lesions separated by normal skin: yes, 0 points, no, 6 points. The four patient-reported symptoms (erythema, edema, pain, purulent discharge) scored 0–3 points each. Additional endpoints, physician global assessment (PGA) (26) and dermatology quality of life index (DLQI) (27) were analyzed.

### Statistical Analysis

Two independent investigators (AJ, AS) extracted primary outcome quantitative data, analyzing mean, standard deviation (SD) and sample size for both the control and intervention groups. In studies where range was mentioned, as a measure of dispersion, it was converted to SD using the formula SD = IQR/1.35, assuming the data followed a normal distribution. Studies were weighted using random effects proposed by DerSimonian and Laird (28). Heterogeneity across RCTs was estimated using the *I*^2^ statistic, whereas a *I*^2^ > 50% was considered significant (28). Publication bias was assessed by visualizing the Begg's funnel plot and Egger's regression analysis and was considered significant at *p* < 0.10 (29). In case of significant publication bias, Duval & Tweedie's Trim & Fill method adjusted the pooled effect size, improving the funnel plot's symmetry. The small number of patients studied meant subgroup analyses could not be performed. GRADE evidence profile was used to evaluate certainty of outcomes, assessed across several domains: study design, risk-of-bias, imprecision, indirectness, inconsistency, publication bias, and strength of effect size (30). Evidence grade was rated from high to very low, with evidence downgraded by one level, where serious concerns pertaining to the aforementioned matrices existed. Meta-analysis was conducted using Comprehensive meta-analysis software (v. 3.0, New Jersey, USA).

## Results

### Search Results

**Figure 1:**
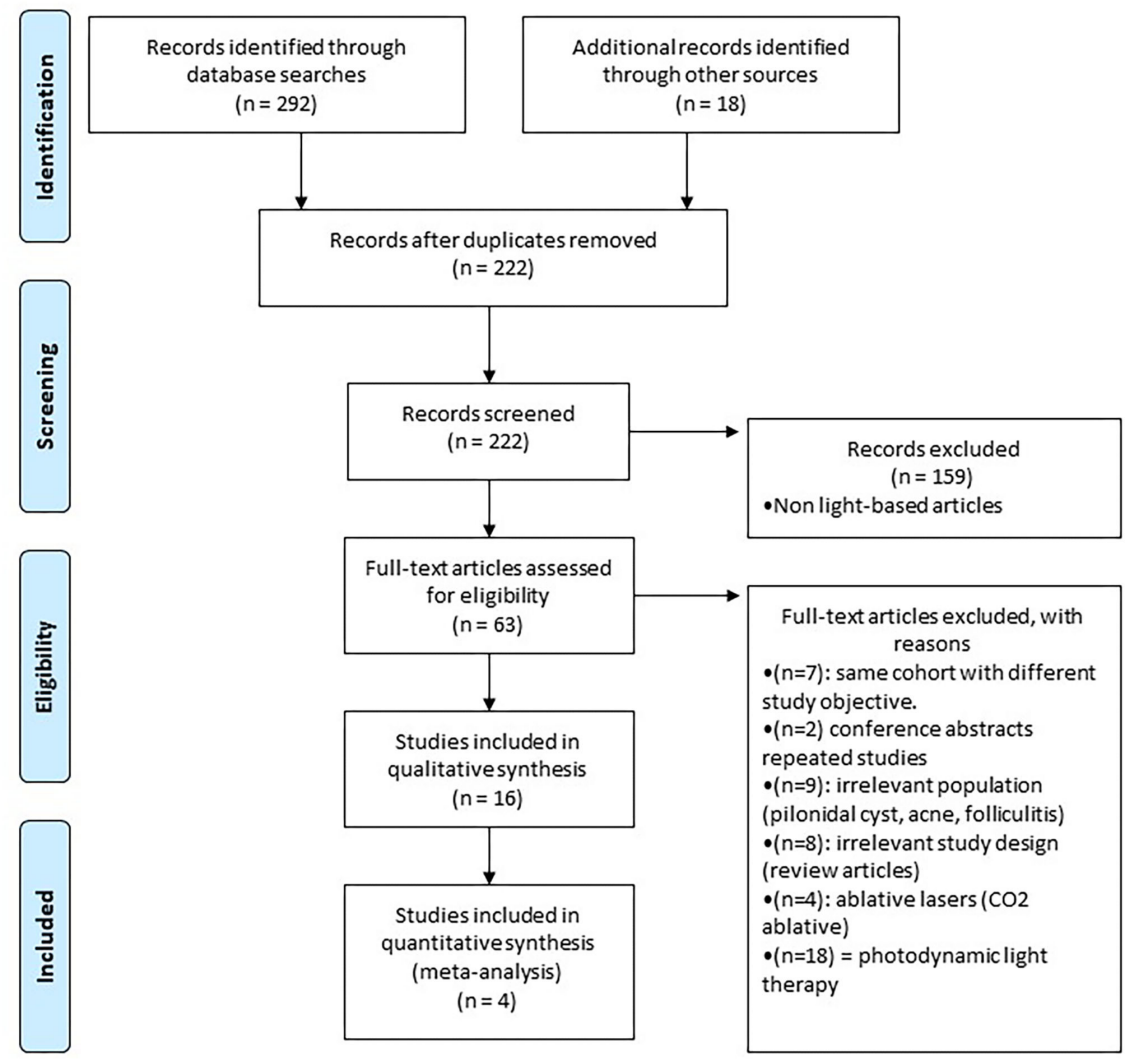
Study flowchart as per the preferred reporting items for systematic reviews and meta-analyses (PRISMA) criteria.

Study design is summarized in a flow diagram (Figure 1). A total of 310 articles were initially identified. After removing duplicates and screening titles, abstracts, and full-texts, 16 articles met the inclusion criteria, which consisted of 5 RCTs and 11 case report/series for a total of 211 unique HS patients. The most commonly investigated laser was Nd:YAG (three RCTs and three case series), followed by IPL (two RCTs and one case series) and Alexandrite (one case report).

### Study and Patient Characteristics

Five RCTs and five valid case reports/series with a total of 206 patients treated with three light-based modalities (IPL, Nd:YAG 1,064 nm and Alexandrite 755 nm) were included. Most patients were females 159 (77%). Table 1 summarizes study patient characteristics.

**Table 1:** Detailed description of patient characteristics in the included studies.

	**Patients characteristics**					**Intervention**			
**Study**	**Sample**	**(M, F); age yrs. mean (range)**	**Fitzpatrick skin type (≤III vs. ≥IV)**	**Hurley stage I/II/III**	**Adjunctive therapies used**	**Laser (type) or IPL**	**Fluence (J/cm^**2**^)/Spot size (mm)/ Pulse duration (ms)**	**Number of sessions**	**Reported outcome result**
**Randomized control trials (RCT)**									
Highton et al. (23)	18	M (*n* = 3), F (*n* = 15); age = 34	NR	II–III	No systemic or topical therapies were allowed 2 weeks prior to and during enrollment	IPL	420 J/cm2/7–10 mm/30–50 ms	2/week × 4 weeks	HS-LASI: decrease by 56% at 3 months, decrease by 44% at 6 months −33% at 12 months on all sites
Mahmoud et al. (15)	22	M (*n* = 3), F (*n* = 19)	NR	II	No systemic or topical therapies were allowed 2 weeks prior to and during enrollment	Nd:YAG 1,064 nm	40–50 J/cm^2^/10 mm/20 ms	1/month × 4 months	HS-LASI: decrease by 72.7% at 6 months all sites
Tierney et al. (31)	22	M (*n* = 3), F (*n* = 19); age = 41 (19–72)	≤III (*n* = 14), ≥IV (*n* = 8)	II–III	No systemic or topical therapies were allowed 2 weeks prior to and during enrollment	Nd:YAG 1,064 nm	40–50 J/cm^2^ (Fitz ≤ III), 25–35 J/cm^2^ (Fitz ≥ IV)/10 mm/20 ms (Fitz ≤ III), 35 ms (Fitz ≥ IV)	1/month × 3 months	HS-LASI: decrease by 65.3% all sites at 3 months
Wilden et al. (32)	43	M (*n* = 12), F (*n* = 31); age = 38 (23–57)	NR	I (*n* = 7), II (*n* = 23), III (*n* = 13)	Patients were not allowed to use topical or systemic therapy during the study (e.g. immunosuppressant, antibiotics, retinoids). Short-time rescue antibiotics, incisions of abscesses and the usage of disinfection were allowed.	IPL	4.4–6.0 J/cm^2^ /8 mm/not avail.	2/week × 24 weeks	DLQI improved by 31% and 46% in patients treated with IPL + RF and RF only
Xu et al. (33)	20	M (*n* = 3), F (*n* = 17); age = 37 (23–54)	≤III (*n* = 11), ≥IV (*n* = 8)	II (*n* = 19)	Topical therapies were allowed but no systemic treatment 2 weeks prior to or during the enrollment	Nd:YAG 1,064 nm	40–50 J/cm^2^ (Fitz ≤ III), 25–35 J/cm^2^ (Fitz ≥ IV)/10 mm/20 ms (Fitz ≤ III), 35 ms (Fitz ≥ IV)	1/month × 2 months	HS-LASI: decrease by 31.6% all sites after 2 sessions
**Case reports/Case series**									
Abdel Azim et al. (34)	20	M (*n* = 9), F (*n* = 11); age= (20–35)	III–IV	I–II	No systemic or topical therapies were allowed 2 weeks prior to and during enrollment	Nd:YAG 1,064 nm	35 J/cm^2^/10 mm/20 ms	1/2 weeks × 4 weeks	PGA score improvement (decrease by 70.68%)
Rucker et al. (35)	20	M (*n* = 3), F (*n* = 17); age = 41 (19–72)	II–IV	II	No systemic or topical therapies were allowed 2 weeks prior to and during enrollment	Nd:YAG 1064 nm	40–50 J/cm^2^ (Fitz ≤ III), 25–35 (Fitz ≥ IV)/10 mm/20 ms (Fitz ≤ III), 35 ms (Fitz ≥ IV)	1/month × 3 months	HS-LASI: decrease in 20.5%
Theut et al. (20)	25	F (*n* = 25); age = 39.24 (16–63)	≤III (*n* = 23), ≥IV (*n* = 2)	I (*n* = 5), II (*n* = 19), III (*n* = 1)	Two patients were on metformin, 16 patients on topical risocinol and 11 patients were on topical clindamycin and systemic tetracycline.	IPL	18–34 J/cm^2^/20 mm or 100 mm/not avail.	1–10 sessions/ 4–6 weeks	13/25 patients had a reduction in disease activity
Tsai et al. (36)	1	M/19	IV	II	No topical or systemic therapies.	Alexandrite 755 nm	22–24 J/cm^2^/18 mm/not avail.	1/month × 3 months	Improvement in pain and discharge
Vossen et al. (37)	15	M (*n* = 10), F (*n* = 5); age = 34.1 ± (10.1)	≤III (*n* = 15)	I	Three out of the 15 patients were on clindamycin 300 mg twice daily and rifampicin 600 mg once daily, minocy-cline 100 mg once daily, and acitretin 25 mg once daily, none of these treatments statistically affected the study outcomes).	Nd:YAG 1,064 nm	30–0 J/cm^2^/7–12 mm/20–40 ms	1/month × 6 months	Decrease number of monthly flares

*IPL, intense pulse light; Nd:YAG, neodymium-doped yttrium aluminum garnet; HS-LASI, hidradenitis suppurativa lesion, area, and severity index; PGA, physician global assessment*.

### Quality Assessment

Five included RCTs were rated as having overall “low risk-of-bias” using Cochrane risk-of-bias two tool, and both investigator evaluations were concordant. Certainty of evidence was rated low due to imprecision and inconsistency noted in outcome as per the GRADE evidence profile (Table 2). Five of 11 case report/series were evaluated as valid and were included in the study (Supplementary Table 1).

**Table 2:** GRADE evidence.

**Certainty assessment**							**# of patients**		**Effect**		**Certainty**	**Importance**
**# of studies**	**Study design**	**Risk of bias**	**Inconsistency**	**Indirectness**	**Imprecision**	**Other considerations**	**[intervention]**	**[comparison]**	**Relative (95% CI)**	**Absolute (95% CI)**		
**HS-LASI (assessed with: HS-LASI scale)**												
3	Randomized control trials	Not serious	Serious^a^	Not serious	Serious^b^	Strong association	53	53	-	SMD **0.99 SD higher** (0.28 higher to 1.71 higher)	⊕⊕○○ LOW	CRITICAL

a *There was evidence for significant heterogeneity in reporting of HS-LASI scores; I squared= 65.37%*.

b *A wide confidence interval*.

*CI, confidence interval; SMD, standardized mean difference*.

### Neodymium-Doped Yttrium Aluminum Garnet Laser (ND-YAG)

The Nd:YAG settings used by all three RCTs and 3 case series were 25–60 J/cm^2^ fluence with 10 mm spot size and 20–35 s pulse duration. Two passes were done over inflamed lesions and one over unaffected skin, with treatments every 4–6 weeks (15, 31, 33–35, 37). Lower energy and higher pulse duration were applied in darker phototype skin (Fitzpatrick IV–VI) HS patients.

In the largest RCT of 22 patients treated with Nd:YAG, the percentage change in HS-LASI score after 3 months was −65.3% averaged over all anatomic sites, with the inguinal region having the greatest reduction by −73.4%, followed by −62.0% for the axillary region and −53.1% for the inframammary region (31).

Disease severity before and after use of Nd:YAG was rated on a numerical rating scale (NRS) ranging from 0 (no suffering) to 10 (extreme/unbearable suffering). Fourteen months after 8 to 10 monthly Nd:YAG sessions, revealed severity being reduced from NRS 6.4 ± 2.8 to NRS 3.6 ± 3.5 (*p* = 0.010) in a case series of 25 patients (37). This was a patient-based survey without physician assessment of outcomes. Hence, responses were subject to recall bias and possibly were impacted by the fluctuating nature of HS. Treated patients reported a 50% reduction in the number of flares and higher satisfaction after treatment completion compared to before Nd:YAG (*p* = 0.019). Additionally, 2 case series of 20 patients each reported improvement in PGA and HS-LASI respectively in all anatomical sites (34, 35). Patient follow-up was only 3 months, which is considered relatively short to assess improvement.

### Intense Pulsed Light

An RCT of 17 patients found that twice-weekly IPL for 4 weeks at 420 nm, 7–10 J/cm^2^, 30–50 ms (assessed at 12 months) significantly improved HS, with a 33% reduction in HS-LASI score (23). Another RCT of 43 patients compared IPL alone (three passes of 420–1200 nm, 4.4–6 J/cm^2^ and 8 ms) to IPL with radiofrequency (RF), and reported that those receiving IPL plus RF experienced improvement in lesion count and DLQI of 44% (*p* = 0.040) at week 12 and 66% (*p* = 0.014) at week 24 compared to the IPL alone (32).

In a case series of 25 patients, a decrease in number of flares and hair reduction occurred after 1–10 sessions every 4–6 weeks with IPL (18–34 J/cm^2^/20 or 100 ms) (20). Patients were mostly Fitzpatrick II–III skin type with the exception of two HS patients (Fitzpatrick type IV) with Hurley I/II, who received four sessions of IPL (500 nm and 550 nm, 9 J/cm^2^, 5–10 ms) at intervals of 15–20 days (18). Both experienced complete resolution of the inflammatory, painful components of HS at 3 months follow up.

### Alexandrite Laser

Our systematic review found no RCTs and only two case reports and one case series that investigated the use of Alexandrite laser for HS. These included a total of 4 HS patients with Hurley stage II disease and Fitzpatrick skin phototype II-III (16, 17, 36). Only one case report met inclusion criteria for this review (36). The setting used in all three studies was a wavelength of 755 nm (15–35 J/cm^2^, 5–28 ms) with one session per 4 weeks. In one patient with Hurley stage III disease, the reported outcome of stopping oral antibiotic was provided without accompanying assessment of severity (16). In the other case (36), pain assessment was performed after only one session of Alexandrite, which is too early to assess treatment efficacy. Furthermore, the patient was on tetracycline for facial acne concomitantly, which is known to have a positive effect on HS and can be a confounder (11).

### Meta-Analysis

Out of the five RCTs, three were included in the meta-analysis. These three employed the modified HS-LASI scale, as the measure of primary outcome (15, 23, 31, 33). One study did not provide enough statistical information for meta-analysis. Hence, only a qualitative assessment was performed (23). Another study measured primary lesion count and DLQI scores as outcomes for efficacy of laser treatment in patients with HS: due to a lack of a common reporting scale it was not included in the meta-analysis (32). Out of the studies included in the meta-analysis, one presented treatment effect size data for participants after splitting them into one group with lesions in axilla and one with lesions in the groin, evaluating them as separate treatment groups (33).

In three studies with valid quantitative data, half intervention/half control study design was employed, with a total sample size of 106 patients with HS. Significant statistical heterogeneity in reporting of HS-LASI existed in these RCTs, where *I*^2^ was measured at 65.37% (*P* = 0.03, *Q* = 8.66). Therefore, we used random effects for weighting them.

**Figure 2:**
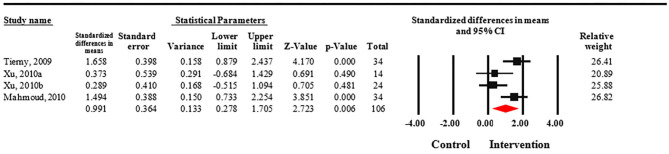
Forest plot of standardized mean differences and confidence intervals of HS-LASI for HS patients treated with Nd:YAG laser compared to controls. It demonstrates that Nd:YAG laser treated patients (*n* = 58) had significantly improved HS-LASI scores compared to the control group.

**Figure 3:**
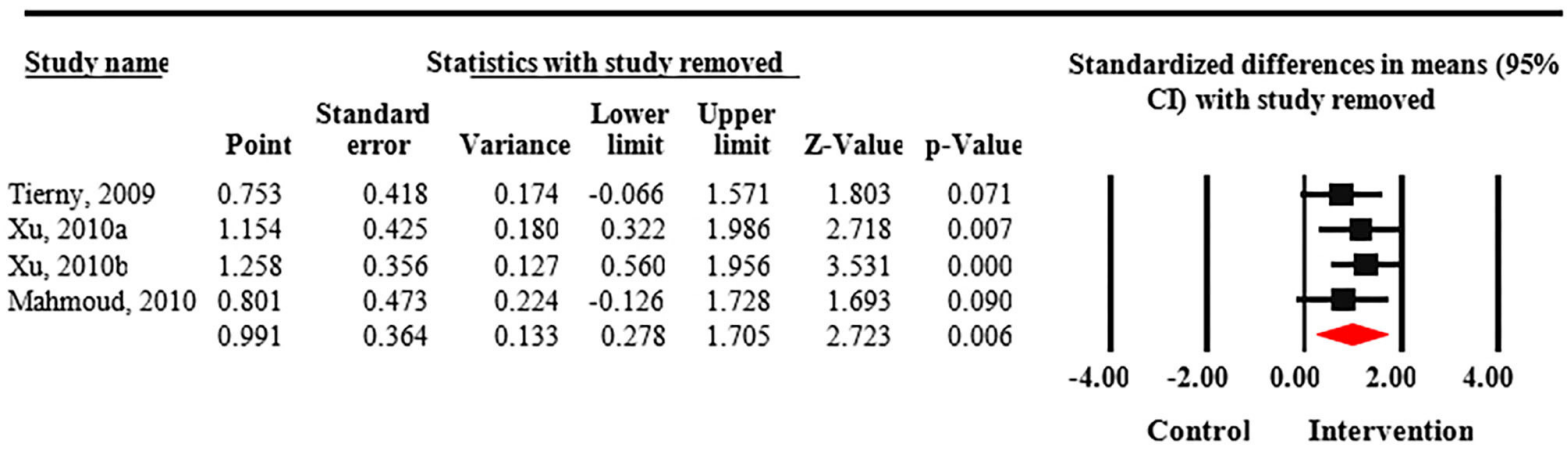
Sensitivity analysis of included randomized control trials. Sensitivity analysis shows non-significant change in pooled effect size pertaining to laser therapy in Hidradenitis suppurativa (HS).

**Figure 4:**
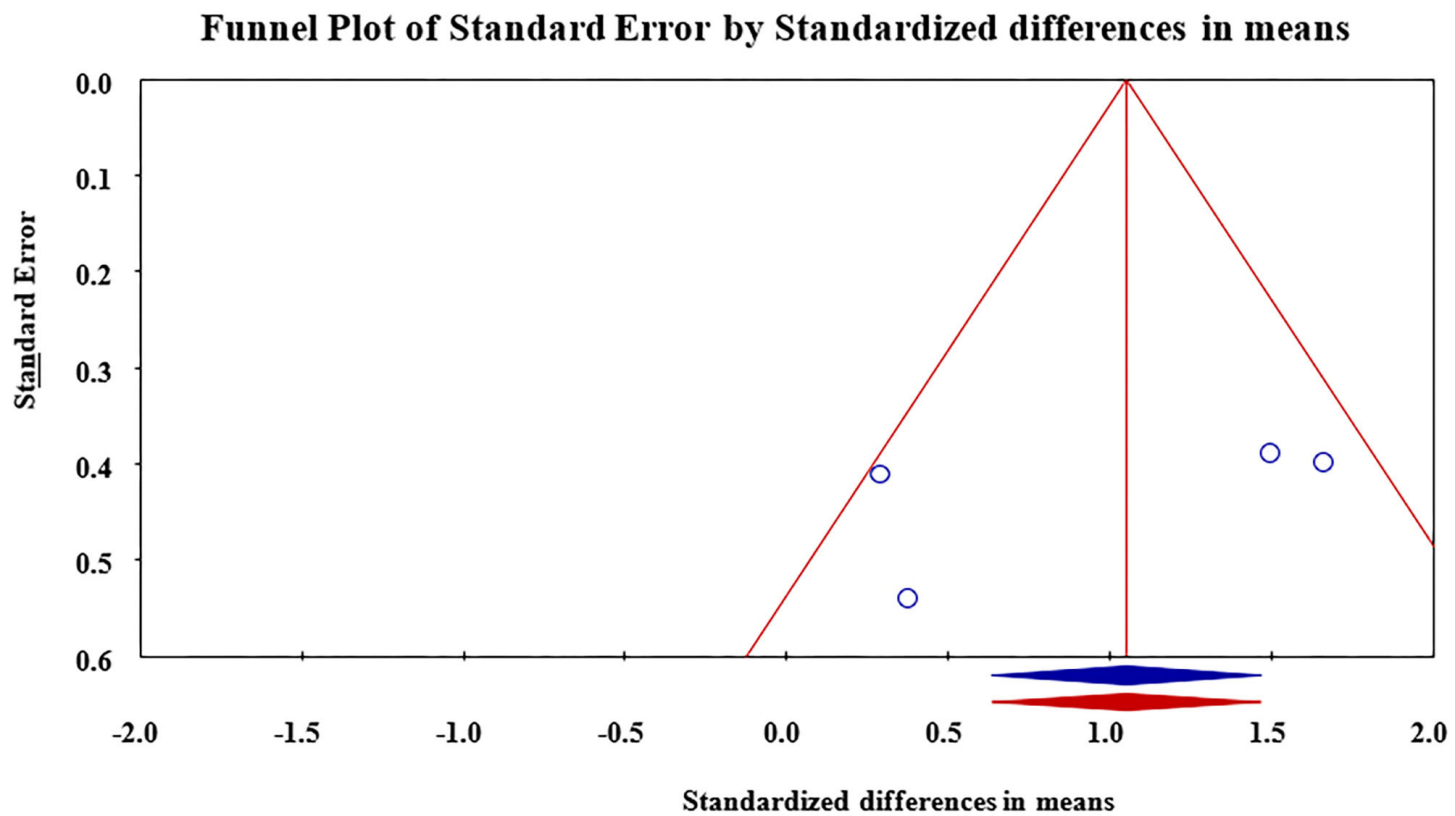
Funnel plot for assessment of publication bias in reporting of outcomes. Publication bias was found to be non-significant.

Meta-analysis revealed that treatment with Nd:YAG laser (58 patients) significantly improved HS-LASI scores compared to the control group with a standardized mean difference (SMD) of 0.99 (95% CI: 0.28 to 1.71, *p* = 0.006) (Figure 2). Sensitivity analysis showed non-significant change in pooled effect size pertaining to laser therapy in HS (Figure 3). There was no evidence of publication bias in this outcome (Figure 4). Egger's regression model was non-significant (B = −6.99, *P* = 0.42).

No unifying outcome was reported using case reports/series. Therefore, results could not be compared by statistical analysis, and only a qualitative assessment could be performed. For the RCTs, the scoring system of HS-LASI was used by 3/5 RCTs and the meta-analysis performed for those modalities had a common reported outcome (38).

## Discussion

Non-ablative light-based therapies targeting the hair follicle and/or water in the dermis can be considered as useful treatment options for patients with HS (39). This mechanism of action is particularly interesting given the role of follicular inflammation in HS pathogenesis. The use of long pulsed Nd:YAG laser resulted in significant improvement in HS lesions compared to the controls (95% CI: 0.28 to 1.71). Analysis of the Alexandrite laser 755 nm and IPL 420 nm also demonstrated improvement in clinical severity, however, given the lack of a uniform reporting scale, these results could not be compared quantitatively through a formal meta-analysis.

One of the possible reasons for Nd:YAG being the most commonly investigated hair removal device in HS is its higher efficacy and safety profile in darker skin patients given the higher likelihood of these individuals being affected by the disease (40). It is yet to be proven whether the earlier use of non-ablative light-based therapies such as Nd:YAG in HS can actually alter the natural history of the disease or delay the progression from Hurley I to stages II–III. Our report highlights the need for larger RCTs to assess the effectiveness of non-ablative lasers.

One of the most significant limitations to recommending routine use of non-ablative light-based therapies remains the price. Importantly, given that non-ablative light devices are costly, not covered by most insurance plans in North America, and that multiple sessions are required, confirming their effectiveness in well-designed randomized trials prior to incorporating them into treatment algorithms remains essential. Future studies should examine dose-response effect and the number of sessions required for significant disease improvement and clinical end results in order to determine cost-effectiveness.

The assessment of effective hair removal is different in HS from other cosmetic treatments since the ultimate goal is to reduce the follicular load that triggers the inflammatory process rather than achieving a hairless skin. Hence, we and others emphasize the use of the modified HS-LASI measure that incorporates the patient's symptoms with the physical examination, when reporting efficacy of laser/IPL use in HS to facilitate future comparisons between studies (38).

## Limitations and Strengths

This is the first systematic review specifically conducted to investigate the role of non-ablative light-based therapies in treating HS. The study's strengths include the use of the PRISMA guidelines and an extensive search including five databases with no restrictions on language, publication date, or study design. Additionally, all studies included in this systematic review were evaluated for quality using published quality assessment tools. Due to the small number of included studies and small sample size of patients overall, a meta-analysis could not be conducted for IPL and for Alexandrite laser. Given the lack of high-quality studies, RCTs and observational studies, firm conclusions about the efficacy and effectiveness of IPL and Alexandrite laser could not be drawn. Finally, the lack of common reporting scales, especially in case reports and case series, limited the ability to draw conclusions.

## Conclusions

Our meta-analysis of Nd:YAG laser in HS patients suggests significant improvement in HS-LASI scores. For IPL, evidence is limited, but suggests improvement in HS-LASI and DLQI scores. For Alexandrite laser, evidence precludes conclusions. Given small sample sizes and inconsistent reporting scales, larger RCTs are required to better determine the efficacy of these modalities in treating HS.

## Data Availability Statement

Publicly available datasets were analyzed in this study. This data can be found at: the data from respective papers can be accessed through www.pubmed.gov.

## Ethics Statement

Since open source data were used for this study ethics review was not required for this systematic review and meta-analysis.

## Author Contributions

AJ and AS searched literature and analyzed included studies. AJ, AS, JR, EN, EO'B, DB, and IL—analyzed data. AJ, AS, and AB—prepared figures. AJ, AS, JR, EN, EO'B, DB, and IL—wrote the paper. JR, EO'B, DB, and IL—supervised the study. All authors contributed to the article and approved the submitted version.

## Conflict of Interest

IL holds research grants from Novartis, Bristol Myers Squibb, and Merck Inc. IL participated in advisory boards for Novartis, Janssen, Galderma, Bristol Myers Squibb, and Bausch Health. EN holds research grants from Novartis, Eli Lilly, and Sanofi Inc. EN participated in advisory boards for Novartis, Sanofi, Eli Lilly, Leo Pharma, Bausch Health, and Abbvie. EN delivered lectures for Leo Pharma and Bausch Health. EO'B holds a research grant from Galderma Inc. EO'B is a member of the board and gave lectures for Abbvie Inc. JR has served as a consultant for Biogen Inc. The remaining authors declare that the research was conducted in the absence of any commercial or financial relationships that could be construed as a potential conflict of interest.
