# Supplementary material for: The Efficacy and Effectiveness of Non-ablative Light-Based Devices in Hidradenitis Suppurativa: A Systematic Review and Meta-Analysis
Source: Front Med (Lausanne). 2020 Nov 3;7:591580. doi: 10.3389/fmed.2020.591580 (PMC7670045; doi:10.3389/fmed.2020.591580)
Supplement: Supplementary file 1 [file Table_1.docx]

| **Study** | **D1** | **D2** | **D3** | **D4** | **D5** | **Overall** |
| --- | --- | --- | --- | --- | --- | --- |
| ***Highton 2011*** | Low | Low | Low | Low | Low | Low |
| ***Mahmoud 2010*** | Low | Low | Low | Low | Low | Low |
| **Tierney 2009** | Low | Low | Low | Low | Low | Low |
| **Wilden 2019** | Low | Low | Low | Low | Low | Low |
| **Xu 2010** | Low | Low | Low | Low | Low | Low |

**Supplementary Table 1.** Cochrane risk of bias assessment v2 of included randomized clinical trials (RCT).

| **Study** | **Design** | **Q1** | **Q2** | **Q3** | **Q4** | **Q5** | **Q6** | **Q7** | **Q8** | **% yes** | **Validity** |
| --- | --- | --- | --- | --- | --- | --- | --- | --- | --- | --- | --- |
| 1. Azim (2018) | Case report | Y | Y | Y | N | N | N | Y | Y | 62.5 | Valid |
| 1. Chan (2013) | Case report | N | Y | N | N | N | N | Y | N | 25 | Not valid |
| 1. Downs (2004) | Case report | N | Y | N | N | N | N | N | N | 12.5 | Not valid |
| 1. Jain (2012) | Case series | N | Y | N | N | N | N | Y | Y | 37.5 | Not valid |
| 1. Piccolo (2014) | Case series | N | Y | N | N | N | N | N | Y | 25 | Not valid |
| 1. Rucker (2009) | Case series | N | Y | Y | Y | N | N | Y | Y | 62.5 | Valid |
| 1. Sehgal (2011) | Case report | N | Y | N | N | N | N | Y | Y | 37.5 | Not valid |
| 1. Sivaramakrishnan (2013) | Case report | N | Y | N | N | N | N | N | Y | 25 | Not valid |
| 1. Theut (2018) | Case series | N | Y | Y | Y | N | Y | Y | Y | 75 | Valid |
| 1. Tsai (2014) | Case report | N | Y | Y | N | N | N | Y | Y | 50 | Valid |
| 1. Vossen (2018) | Case series | N | Y | Y | N | N | N | Y | Y | 50 | Valid |

**Supplementary Table 2.** Risk of bias assessment of included case reports/series. Y:yes, N: no. Case report/series was considered valid when y% is ≥ 50%.

Q1. Does the patient(s) represent(s) the whole experience of the investigator (center) or is the selection method unclear to the extent that other patients with similar presentation may not have been reported?

Q2. Was the exposure adequately ascertained?

Q3. Was the outcome adequately ascertained?

Q4. Were other alternative causes that may explain the observation ruled out?

Q5. Was there a challenge/rechallenge phenomenon?

Q6. Was there a dose–response effect?

Q7. Was follow-up long enough for outcomes to occur?

Q8. Is the case(s) described with sufficient details to allow other investigators to replicate the research or to allow practitioners make inferences related to their own practice?
